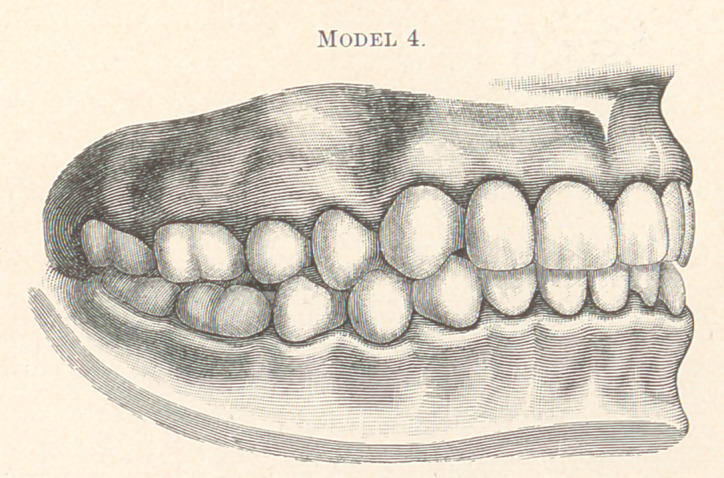# A Case of Drawing the Lower Jaw Forward

**Published:** 1894-06

**Authors:** H. E. Cutter

**Affiliations:** Cambridge, Mass.


### International Dental Journal.

Vol. XV. June, 1894. No. 6.

### Original Communications.[1]

### A CASE OF DRAWING THE LOWER JAW FORWARD.[2]

BY H. E. CUTTER, D.D.S., CAMBRIDGE, MASS.

Mr. President and Gentlemen,—It was with some hesitancy that I accepted your invitation to prepare the paper which I bring before you. The subject is "A Case of drawing the Lower Jaw Forward."

The patient was a girl eleven years of age. The upper arch was narrow and pointed, with the front teeth very prominent. The lower arch was regular, but the front teeth were elongated in comparison with the bicuspids, so that their cutting-edges almost touched the gum behind the upper incisors. The upper centrals projected fully a quarter of an inch beyond the lower teeth, as shown in Model 1. The upper and lower cuspids, however, almost touched each other. When the mouth was at rest, the lips did not cover the front teeth, so that the face had an unpleasant expression. The profile was even more uncomely, for, besides the projecting teeth, the chin was receding, and seriously marred what would otherwise have been a fine face.

I think that it is important to make a careful study of the

[1] The editor and publishers are not responsible for the views of authors of papers published in this department, nor for any claim to novelty, or otherwise, that may be made by them. No papers will be received for this department that have appeared in any other journal published in the country.

[2] Read before the American Academy of Dental Science, February 7, 1894.

general outline of a patient's face, as well as of the teeth, before beginning any corrective treatment. And we should take into consideration the teeth and facial expression of the parents of the patient, in order that we may know what is likely to be the natural development in the child. I think that, if this were more frequently done, we should have more satisfactory results. By recognizing a family tendency to a deformity at an early age, a simple method can often be successfully employed to prevent its development. Another patient might have a set of teeth very similar to the one before us, and yet an entirely different treatment be required. In the one case the deformity might be due to a receding lower jaw, but in the other to a projecting upper jaw. The first and most important question in cases of this kind is to decide at the outset whether it is the upper or the lower jaw which requires treatment, for upon this, success or failure largely depends.

Before undertaking a case of this character, one should first satisfy himself that the patient is one who will willingly undergo some unpleasant requirements. For there is occasionally a person who is very anxious to undergo treatment, and for a time he faithfully follows the instructions given; but gradually he becomes indifferent and careless, and the treatment of the patient has to be abandoned.

In the case before us it was evident that the lower jaw could not be carried forward without first expanding the upper arch. This was done by means of a thin rubber plate, into which was vulcanized a German silver jack-screw. To hold the width which was thus gained, and at the same time to flatten the arch, another plate was made. A spring German silver wire was bent around close to the outside of the teeth, with its ends embedded in the plate at the sides. From time to time the wire was bent so as to bear hard on the mesial edges of the centrals, and thus the arch was gradually flattened. The result is seen in Model 2.

This being done, the next step was a more difficult one, which was to lengthen the bite and at the same time draw the lower jaw forward. The plate that I next made was like the one which Dr. Hamilton used in a case which he once described before this society. This plate was made thick over the bicuspids and first molars and behind the front teeth. Deep depressions were made in the plate a little forward of the places where the cusps of the lower teeth would naturally touch the plate. The result was that the patient began to carry the lower jaw forward a little, so that the teeth would enter these depressions.

Model 1.

Model 2.

Model 3.

Model 4.

I found, however, that a plate which covered the bicuspids and first molars prevented these teeth, both in the upper and lower jaws, from elongating and forming a new articulation to correspond with the lengthened bite. Therefore I made another plate for the upper arch. This one was thickened only behind the front teeth, where depressions were made to receive the points of the lower incisors. No other teeth of the lower jaw were allowed to touch any part of the plate. This plate was held firmly in place by wire clasps encircling the sixth-year molars. There was also attached to this plate a wire which passed around the outside of the front teeth to keep them in the flattened position which they had assumed.

In making this plate much care was required to have the depressions in just the right places and of exactly the proper depth. A wax and paraffine base-plate was fitted to the plaster model of the upper arch. To the part where the plate was to be thickened soft yellow wax was then added. While the wax was soft the base-plate was inserted in the mouth, and the patient told to throw the lower jaw forward and bite into the wax. I thus determined how much the jaw was then to be carried forward and the bite lengthened. This was an important question, for a slight variation at this point might have produced an unfortunate result. I made several plates of this character, as the amount to be gained had to be gradually accomplished. Model 3 was made while this work was being done.

When I took the case, but one twelfth-year molar had begun to appear; when the work was completed, all four of these molars had erupted and interlocked with each other. The result was that the patient could comfortably bring her jaws together only as they had been newly related.

All that then remained was for the bicuspids and sixth-year molars to complete their articulation, which they are now doing, as is shown in Model 4.

It was necessary to carry the lower jaw forward while the twelfth-year molars were erupting, as the retaining of the jaw in the new position depended entirely on the articulating of these teeth. Unless the operation had been undertaken at just this time, it is doubtful if it could have been accomplished. I think that it would not have been possible to secure the retaining of the jaws in the relation they now are had the attempt been made at an earlier or a later time.

## Figures and Tables

**Model 1. f1:**
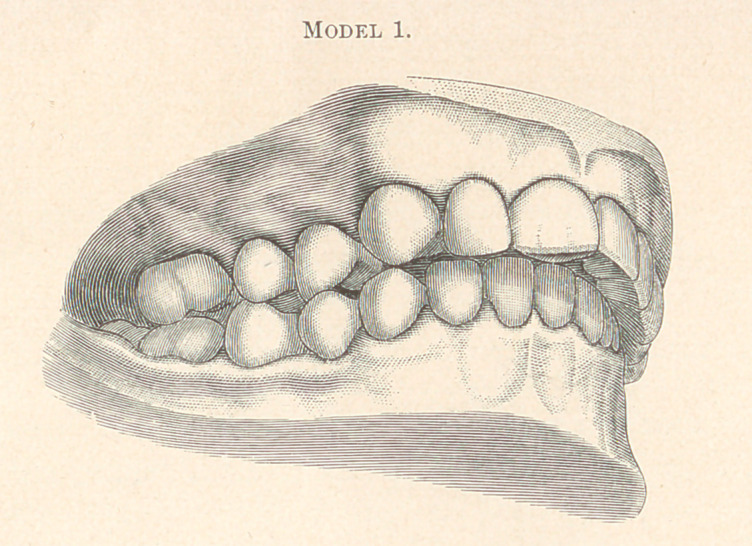


**Model 2. f2:**
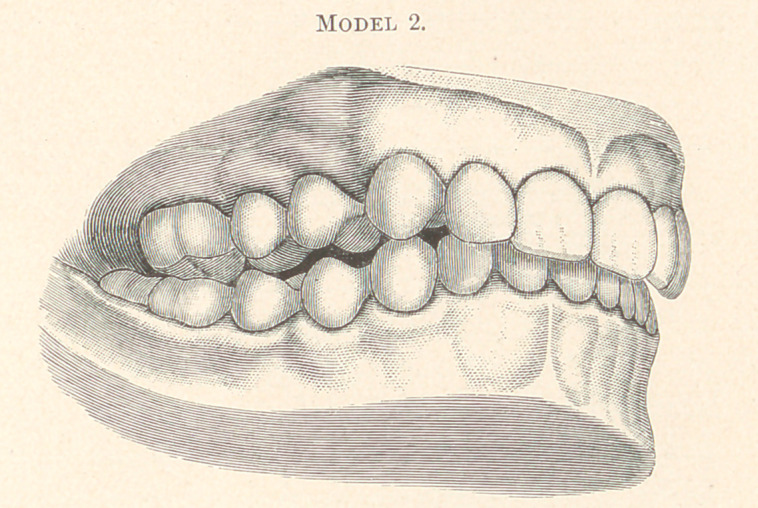


**Model 3. f3:**
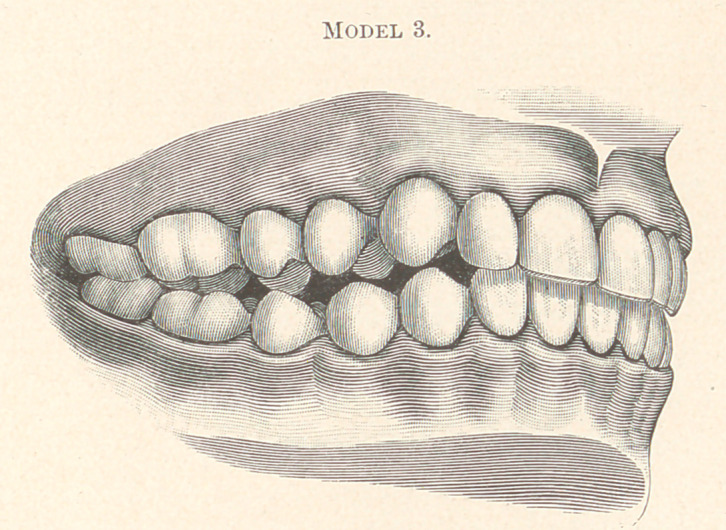


**Model 4. f4:**